# Development and Verification of a Hypoxic Gene Signature for Predicting Prognosis, Immune Microenvironment, and Chemosensitivity for Osteosarcoma

**DOI:** 10.3389/fmolb.2021.705148

**Published:** 2022-01-05

**Authors:** Fengfeng Wu, Juntao Xu, Mingchao Jin, Xuesheng Jiang, Jianyou Li, Xiongfeng Li, Zhuo Chen, Jiangbo Nie, Zhipeng Meng, Guorong Wang

**Affiliations:** ^1^ Department of Orthopedics and Rehabilitation, Huzhou Central Hospital, Affiliated Central Hospital of Huzhou University, Zhejiang University Huzhou Hospital, Huzhou, China; ^2^ Department of Orthopedics, Huzhou Traditional Chinese Medicine Hospital, Affiliated to Zhejiang Chinese Medical University, Huzhou, China; ^3^ Department of Orthopedics, Huzhou Central Hospital, Affiliated Central Hospital of Huzhou University, Zhejiang University Huzhou Hospital, Huzhou, China; ^4^ Department of Anesthesiology, Huzhou Central Hospital, Affiliated Central Hospital of Huzhou University, Zhejiang University Huzhou Hospital, Huzhou, China

**Keywords:** osteosarcoma, prognosis, hypoxia, gene signature, immune microenvironment, nomogram

## Abstract

**Objective:** Hypoxic tumors contribute to local failure and distant metastases. Nevertheless, the molecular hallmarks of hypoxia remain ill-defined in osteosarcoma. Here, we developed a hypoxic gene signature in osteosarcoma prognoses.

**Methods:** With the random survival forest algorithm, a prognostic hypoxia-related gene signature was constructed for osteosarcoma in the TARGET cohort. Overall survival (OS) analysis, receiver operating characteristic (ROC) curve, multivariate cox regression analysis, and subgroup analysis were utilized for assessing the predictive efficacy of this signature. Also, external validation was presented in the GSE21257 cohort. GSEA was applied for signaling pathways involved in the high- and low-risk samples. Correlation analyses between risk score and immune cells, stromal/immune score, immune checkpoints, and sensitivity of chemotherapy drugs were performed in osteosarcoma. Then, a nomogram was built by integrating risk score, age, and gender.

**Results:** A five-hypoxic gene signature was developed for predicting survival outcomes of osteosarcoma patients. ROC curves confirmed that this signature possessed the well predictive performance on osteosarcoma prognosis. Furthermore, it could be independently predictive of prognosis. Metabolism of xenobiotics by cytochrome P450 and nitrogen metabolism were activated in the high-risk samples while cell adhesion molecules cams and intestinal immune network for IgA production were enriched in the low-risk samples. The low-risk samples were characterized by elevated immune cell infiltrations, stromal/immune scores, TNFRSF4 expression, and sensitivity to cisplatin. The nomogram accurately predicted 1-, 3-, and 5-years survival duration.

**Conclusion:** These findings might offer an insight into the optimization of prognosis risk stratification and individualized therapy for osteosarcoma patients.

## Introduction

Osteosarcoma represents the main primary bone malignancy, occurring in 2.4% of children and adolescents (Yang et al., 2020). This disease typically presents in distal femoral metaphysis, followed by the upper extremities, pelvis, and the like (Kelley et al., 2020). Surgical resection and chemotherapy distinctly prolong survival duration of patients with local osteosarcoma. Non-metastatic patients reach the 60–70% of the 5-years survival rate following diagnosis (Cao et al., 2020). However, osteosarcoma is characterized by high metastatic and recurrent risks. For metastatic or relapsed subjects, the 5-years survival rate is only 20% (Shi et al., 2020). It is urgent to probe biomarkers for assisting clinicians to predict survival outcomes as well as offer a basis upon personalized medicine.

The intratumoral hypoxia is a noticeable feature in solid tumors, and hypoxia is in relation to aggressive phenotypes and metastases (Song et al., 2020). For osteosarcoma, hypoxia is involved in facilitating excessive tumor proliferative and invasive capacities through inducing serial molecular reactions to promote the formation of the neoplastic microenvironment (Chen et al., 2020). Under hypoxic conditions, various genes can be activated in osteosarcoma cells. For instance, hypoxia induces chemoresistance through down-regulation of SKA1 expression in osteosarcoma (Ma et al., 2017). Hypoxia-induced WSB1 accelerates invasion and metastasis of osteosarcoma cells (Cao et al., 2015). Nevertheless, no hypoxic gene signature has been established yet for osteosarcoma patients’ prognosis. Transcriptomic analysis may uncover the entire expression patterns as well as identify cancer-related markers. Here, this study effectively developed a robust prognosis-related hypoxic gene signature for osteosarcoma, which might provide a basis for an in-depth understanding of risk stratification and therapeutic effects of osteosarcoma patients.

## Materials and Methods

### Data Collection

**FIGURE 1:**
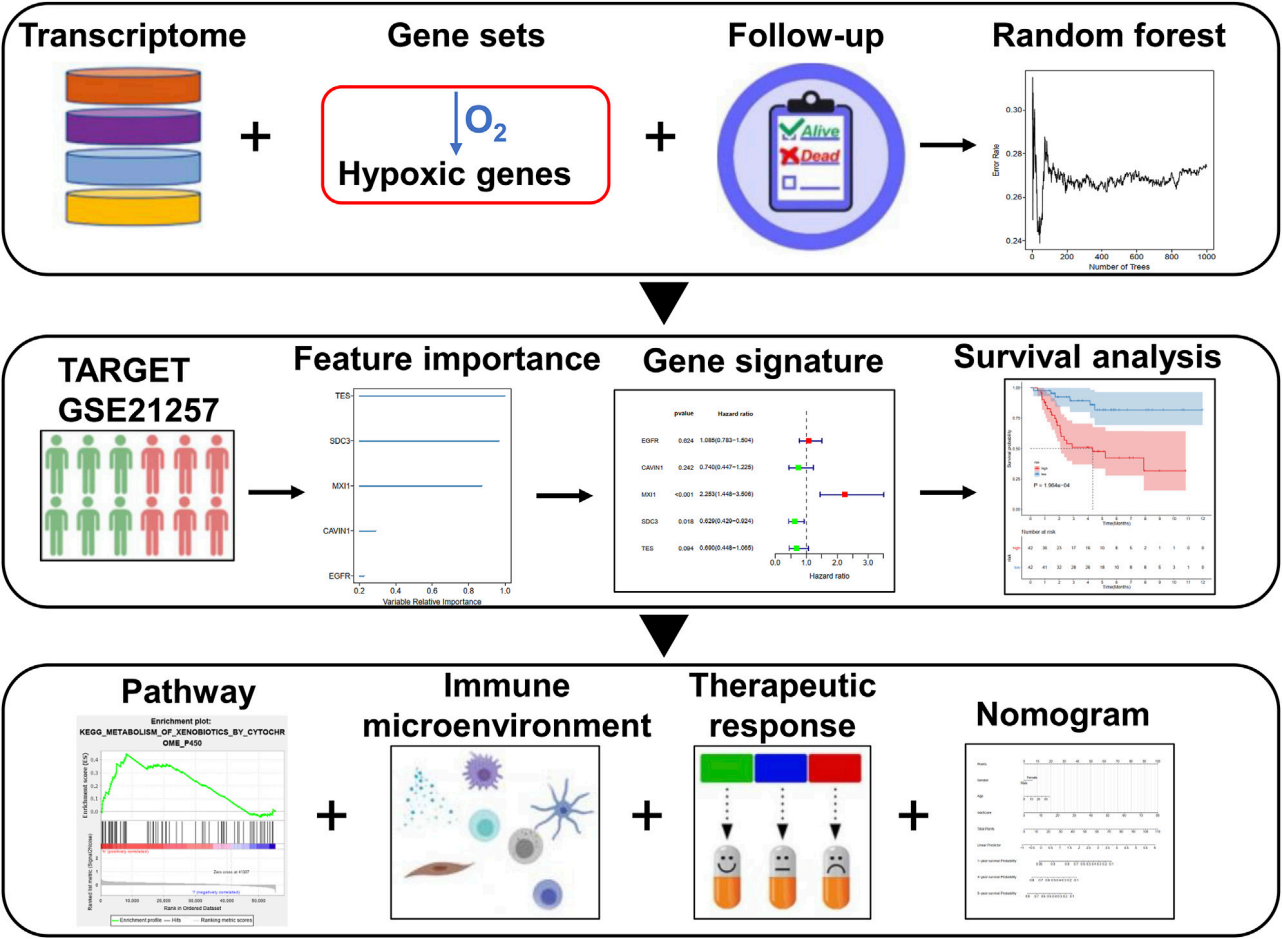
Schematic diagram of the workflow of this study.

The gene expression profiles of 84 cases of osteosarcoma patients with complete follow-up information were downloaded from the Therapeutically Applicable Research to Generate Effective Treatments (TARGET; https://ocg.cancer.gov/programs/target) database, which were utilized as the training set. The GSE21257 dataset containing expression profiling of 53 osteosarcoma subjects was retrieved from the Gene Expression Omnibus (GEO) repository (https://www.ncbi.nlm.nih.gov/geo/) (Buddingh et al., 2011), which was used as the external validation set. Genes up-regulated in response to hypoxia were retrieved from the “HALLMARK_HYPOXIA” gene set in the Molecular Signatures database v7.2 with the gene set enrichment analysis (GSEA) software (Subramanian et al., 2005). Figure 1 depicts the workflow of this study.

### Establishment of a Prognostic Gene Signature

Survival-related hypoxic genes with *p* < 0.05 were screened for osteosarcoma samples from the TARGET cohort with the univariate cox regression analyses. Then, the importance of these prognostic genes was ranked using the random survival forest algorithm. The number of Monte Carlo iterations was set as 100 and the number of steps forward was set as 5. The genes with relative importance >0.25 were feature factors. Multivariate cox regression analyses were then presented. The risk scoring model was established as follows: risk score = coefficient of gene 1 * expression of gene 1 + coefficient of gene 2 * expression of gene 2 +…+ coefficient of gene n * expression of gene n. The specimens were classified into high and low risk groups on the basis of the mean value of risk scores. Expression patterns of genes in the signature were visualized into a heat map. Survival status was evaluated between the two groups. Kaplan-Meier curves of overall survival (OS) were then depicted between groups and survival differences were compared via log-rank test. The time-dependent receiver operating characteristic (ROC) was analyzed for examining the sensitivity and specificity of the gene signature for predicting OS. The area under the curve (AUC) was determined for assessing the prognostic accuracy. Furthermore, the AUC of this signature was compared with other gene signatures developed by Wen C. et al. (2020), Wu Z.-l. et al. (2020), Xiao et al. (2020) and Zhang et al. (Yiqi et al., 2020). Univariate and multivariate cox regression analyses were carried out based on risk score, age, and gender as variables. *p* values, hazard ratio (HR) as well as 95% confidence interval (CI) were calculated. Moreover, survival analysis of each gene in this signature was presented for osteosarcoma subjects in the TARGET cohort.

### Subgroup Analysis

Osteosarcoma samples were classified into different subgroups according to age (<16 and ≥16) and gender (female and male). In each subgroup, OS differences were compared between high and low risk samples with Kaplan-Meier curves and log-rank tests.

### GSEA

Osteosarcoma specimens in the TARGET cohort were classified into high and low risk groups. GSEA was carried out to explore the underlying pathways involved in the two groups. The |normalized enrichment score (NES)| > 1.5 and false discovery rate (FDR) < 0.05 were used to determine statistical significance.

### Single-Sample Gene Set Enrichment Analysis

The enrichment scores of activated B cells, activated CD4 T cells, activated CD8 T cells, central memory CD4 T cells, central memory CD8 T cells, effector memory CD4 T cells, effector memory CD8 T cells, gamma delta T cells, immature B cells, memory B cells, regulatory T cells, T follicular helper cells, type 1 T helper cells, type 17 T helper cells, type 2 T helper cells, activated dendritic cells, CD56bright natural killer cells, CD56dim natural killer cells, eosinophils, immature dendritic cells, macrophages, mast cells, MDSCs, monocytes, natural killer cells, natural killer T cells, neutrophils, and plasmacytoid dendritic cells were determined in high and low risk samples from the TARGET cohort with the ssGSEA algorithm.

#### Estimation of Stromal and Immune Cells in Malignant Tumours Using Expression Data

With the ESTIMATE algorithm, stromal and immune scores were inferred in high and low risk samples from the TARGET cohort based on the gene expression profiling (Yoshihara et al., 2013).

### Correlation Between Risk Score and Immune Checkpoint Expression

Pearson’s correlations between risk scores and expression of immune checkpoints including ADORA2A, BTLA, BTNL2, CD160, CD200, CD200R1, CD244, CD27, CD274, CD276, CD28, CD40, CD40LG, CD44, CD48, CD70, CD80 CD86, CTLA4, HAVCR2, HHLA2, ICOS, ICOSLG, IDO1, IDO2, KIR3DL1, LAG3, LAIR1, LGALS9, NRP1, PDCD1, PDCD1LG2, TIGIT, TMIGD2, TNFRSF14, TNFRSF18, TNFRSF25, TNFRSF4, TNFRSF8, TNFRSF9, TNFSF14, TNFSF15, TNFSF18, TNFSF4, TNFSF9, VSIR, and VTCN1 were assessed in the osteosarcoma specimens from the TARGET cohort.

### Assessment of Chemotherapy Drug Sensitivity

Through the Genomics of Drug Sensitivity in Cancer (GDSC) database (www.cancerRxgene.org) (Yang et al., 2013), the IC50 values of chemotherapy drugs (cisplatin, doxorubicin, methotrexate, and paclitaxel) were estimated in each osteosarcoma specimen with the pRRophetic package (Geeleher et al., 2014).

### Nomogram

A nomogram was established by integrating gender, age, and risk score with the “rms” package for osteosarcoma prognoses. The calibration plots were depicted for verifying the accuracy of prediction of 1-, 3-, and 5-years survival. Also, the predictive efficacy of this nomogram was evaluated by time-dependent ROC analysis.

### Quantitative Real-Time Polymerase Chain Reaction

RNA extraction was performed with Trizol and extracted RNA was converted to cDNA utilizing Super Script II cDNA synthesis kit (Beyotime, China). qRT-PCR was carried out utilizing iTaq universal SYBR Green Supermix (BioRad, CA) on the CFX6100 qPCR instrument (BioRad, CA). The primer sequences were as follows: Stearoyl-CoA desaturase (SCD), 5′-TCT​AGC​TCC​TAT​ACC​ACC​ACC​A-3’ (forward), 5′-TCG​TCT​CCA​ACT​TAT​CTC​CTC​C-3’ (reverse); Epidermal Growth Factor Receptor (EGFR), 5′-AGG​CAC​GAG​TAA​CAA​GCT​CAC-3’ (forward), 5′-ATG​AGG​ACA​TAA​CCA​GCC​ACC-3’ (reverse); MAX Interactor 1 (MXI1), 5′-GCG​CCT​TTG​TTT​AGA​ACG​CTT-3’ (forward), 5′-AAT​GCT​GTC​CAT​TCG​TAT​TCG​T-3’ (reverse); Caveolae Associated Protein 1 (CAVIN1), 5′- GAG​GAC​CCC​ACG​CTC​TAT​ATT-3’ (forward), 5′-CCC​CGA​TGA​TTT​TGT​CCA​GGA-3’ (reverse); Testin (TES), 5′-ATG​GGC​TTA​GGT​CAC​GAG​C-3’ (forward), 5′-TCC​CAC​TTT​TCG​ATC​CTC​TTC​A-3’ (reverse); GAPDH, 5′-GGA​GCG​AGA​TCC​CTC​CAA​AAT-3’ (forward), 5′-GGC​TGT​TGT​CAT​ACT​TCT​CAT​GG-3’ (reverse). The relative mRNA expression was calculated with 2^−ΔΔCt^ method.

### Western Blotting

After implementing protein extraction from tissues utilizing RIPA, protein was loaded into SDS-PAGE gel and transferred into polyvinylidene difluoride membranes. Thereafter, the membranes were sealed by 5% BSA blocking buffer as well as incubated with primary antibodies against SCD (1:1,000; ab236868; Abcam, United States), EGFR (1:1,000; ab52894; Abcam), MXI1 (1:1,000; 12360-1-AP; Proteintech, Wuhan, China), CAVIN1 (1:1,000; ab76919; Abcam), TES (1:1,000; 10258-1-AP; Proteintech), and GAPDH (1:1,000; 6,004-1-lg; Proteintech) overnight at 4°C. Afterward, HRP-conjugated anti-rabbit IgG (1:2000; SA00001-2; Proteintech) was applied for hatching the membranes for 2 h. The membranes were exposed to enhanced chemiluminescence (ECL) solution and the gray value of protein bands was quantified with ImageJ software (NIH, United States).

### Cell Culture and Transfection

Human osteoblast hFOB1.19 and two human osteosarcoma cells Saos-2 and U2OS (ATCC, United States) were maintained in DMEM plus 10% heat-inactivated fetal bovine serum (Sigma, United States) and 2% antibiotic-antimycotic solution (Corning, United States) at 37°C in a humidified atmosphere with 5% CO_2_. Specific siRNAs against MXI1 (si-MXI1) and negative control (si-NC) were synthesized by GenePharma (Shanghai, China). In total, 3 × 10^5^ Saos-2 and U2OS cells were seeded onto a 6-well plate, and transiently transfected with 10 nM siRNA utilizing Lipofectamine iMAX kit (Invitrogen, United States). Saos-2 and U2OS cells were collected for RNA extraction following 48 h of incubation with specific siRNAs.

### Colony Formation Assay

In total, 500 Saos-2 and U2OS cells were planted onto a 6-well dish. After culturing for 2 weeks, the colonies were washed by PBS and dyed with a dyeing solution containing 0.1% crystal violet and 20% methanol. Thereafter, the colonies were counted.

### Transwell Assays

Transwell assays were performed for measuring migration and invasion of Saos-2 and U2OS cells utilizing 24-well inserts (8 μm pore, Corning, United States). For migration, Saos-2 and U2OS cells re-suspended in 100 μl serum-free DMEM medium were added to the upper chamber without coating membrane. For invasion, re-suspended Saos-2 and U2OS cells were added to the upper chamber with Matrigel-coated membrane. DMEM plus 600 μl 10% FBS was added to the lower chamber. Migrated or invaded cells from the bottom surfaces were fixed by 4% paraformaldehyde, followed by being stained by 0.1% crystal violet for 20 min. Finally, the cells were investigated and counted under a microscope (Leica, Germany).

### Statistical Analysis

All statistics were carried out by applying the R software (version 3.6.5; https://www.R-project.org) and its packages. Comparisons between two groups were presented via Wilcoxon or Student’s t tests. Multiple comparisons were presented through one- or two-way ANOVA test. *p* values <0.05 were set as statistical significances.

## Results

### Establishment of a Prognostic Hypoxic Gene Signature in Osteosarcoma

**FIGURE 2:**
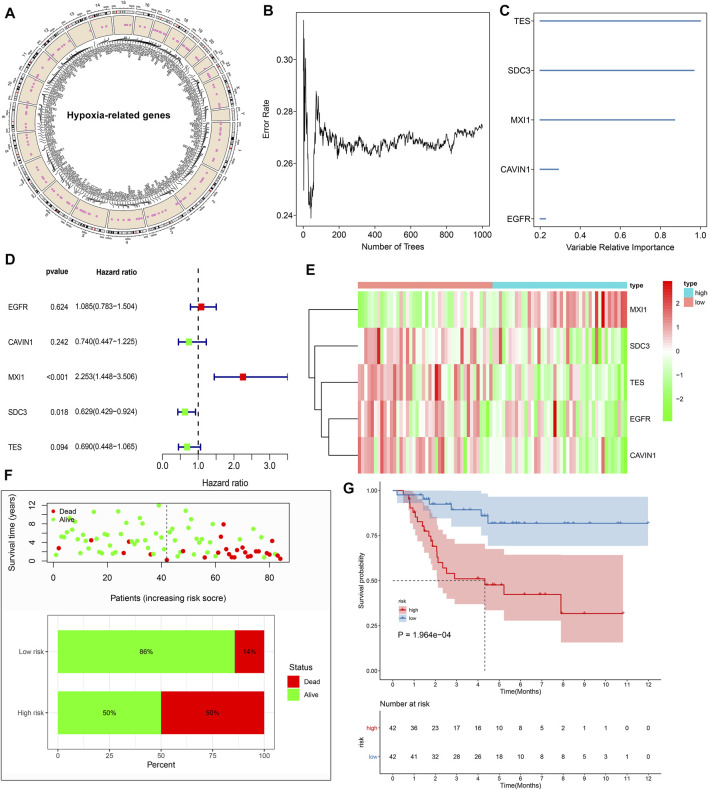
Establishment of a prognostic hypoxic gene signature in osteosarcoma in the TARGET cohort. **(A)** The chromosomal locations of 197 hypoxic genes. **(B)** The relationships between the number of trees and error rates. **(C)** The rank of relative importance of the top five variables. **(D)** Univariate cox regression analysis of TES, SDC3, MXI1, CAVIN1, and EGFR in osteosarcoma. **(E)** Heat map visualizing the up- (red) and down-regulated (green) genes in high- and low-risk groups. **(F)** The distributions and proportions of survival status (alive: green; dead: red). **(G)** Kaplan-Meier curves of OS between high- and low-risk specimens. The differences were compared by log-rank tests.

In total, 197 hypoxic genes were obtained for this study (Supplementary Table S1). The chromosomal locations of these genes are shown in Figure 2A. Through univariate cox regression analysis, 26 hypoxic genes exhibited significant correlations to prognosis of osteosarcoma patients in the TARGET cohort (Table 1). The most important hypoxic genes related to osteosarcoma prognosis were selected using the random survival forest algorithm (Table 2). When the number of classification trees was 5, the error rate was the lowest (Figure 2B). The feature importance ranking of the top five genes is shown in Figure 2C, including TES (chr7: 116210506–116258783), SDC3 (chr1: 30869466–30908758), MXI1 (chr10: 110207605–110287365), CAVIN1 (chr17: 42402449–42423256), and EGFR (chr7: 55019017–55211628). Following multivariate cox regression analysis, a five-hypoxic gene signature was constructed in osteosarcoma (Figure 2D). The scoring formula was as follows: risk score = 0.081751 * EGFR expression + (−0.3013) * CAVIN1 expression +0.812177 * MXI1 expression + (−0.46302) * SDC3 expression + (−0.37064) * TES expression. According to the median value of risk scores, osteosarcoma patients were separated into high- and low-risk subgroups (n = 42). The heat map visualized the expression patterns of TES, SDC3, MXI1, CAVIN1, and EGFR in high- and low-risk specimens (Figure 2E). The proportions of dead patients were separately 50 and 14% in high- and low-risk groups (Figure 2F). Moreover, high-risk scores were indicative of unfavorable survival outcomes of osteosarcoma patients (Figure 2G).

**TABLE 1:** Univariate cox regression analysis for prognosis-related hypoxic genes in the TARGET cohort.

ID	HR	HR.95L	HR.95H	*p*-value
DCN	0.687972	0.532825	0.888294	0.004125
PYGM	1.241066	1.042373	1.477632	0.015259
SCARB1	1.490841	1.101504	2.017792	0.009709
TPD52	1.504467	1.113686	2.032368	0.007776
HMOX1	0.740526	0.559761	0.979666	0.035392
MYH9	0.613777	0.389335	0.967604	0.035574
NDRG1	1.342496	1.001186	1.80016	0.049082
VEGFA	1.440634	1.123444	1.847379	0.00401
LOX	1.778616	1.212237	2.609618	0.003241
STC2	1.653632	1.204877	2.269526	0.001847
FAM162A	1.780223	1.239705	2.55641	0.001785
MXI1	2.248538	1.50109	3.368166	8.49E-05
P4HA1	1.681275	1.192228	2.370927	0.003051
TES	0.588369	0.40146	0.862299	0.006536
CASP6	0.370699	0.17907	0.767399	0.007515
PKLR	1.714786	1.075027	2.735273	0.023597
PPFIA4	1.631697	1.13218	2.351601	0.008646
EGFR	0.725994	0.553052	0.953015	0.021076
PDK1	1.904419	1.347504	2.691504	0.000262
SDC3	0.569725	0.395219	0.821283	0.002569
ANKZF1	1.954758	1.142563	3.344305	0.014429
LALBA	2.839392	1.090585	7.392502	0.032551
EFNA1	1.72147	1.131121	2.619931	0.011246
PFKFB3	1.554082	1.057866	2.28306	0.024664
CAVIN1	0.59111	0.394464	0.885787	0.010845
MAFF	1.565688	1.14174	2.147055	0.005391

**TABLE 2:** The relative importance of prognosis-related hypoxic genes.

Genes	Raw. Importance	Rel. Importance
TES	0.019695	1
SDC3	0.01906	0.967742
MXI1	0.017154	0.870968
CAVIN1	0.005718	0.290323
EGFR	0.004447	0.225806
TPD52	0.003177	0.16129
LOX	0.001271	0.064516
MAFF	0.001271	0.064516
MYH9	0	0
PYGM	−0.00127	−0.06452
NDRG1	−0.00127	−0.06452
PPFIA4	−0.00127	−0.06452
VEGFA	−0.00191	−0.09677
PKLR	−0.00191	−0.09677
LALBA	−0.00191	−0.09677
HMOX1	−0.00254	−0.12903
EFNA1	−0.00254	−0.12903
SCARB1	−0.00381	−0.19355
CASP6	−0.00508	−0.25806
ANKZF1	−0.00508	−0.25806
FAM162A	−0.00572	−0.29032
STC2	−0.00699	−0.35484
P4HA1	−0.00826	−0.41935
PFKFB3	−0.00889	−0.45161
DCN	−0.01398	−0.70968
PDK1	−0.01715	−0.87097

### The Hypoxic Gene Signature Robustly and Independently Predicts Osteosarcoma Prognosis

**FIGURE 3:**
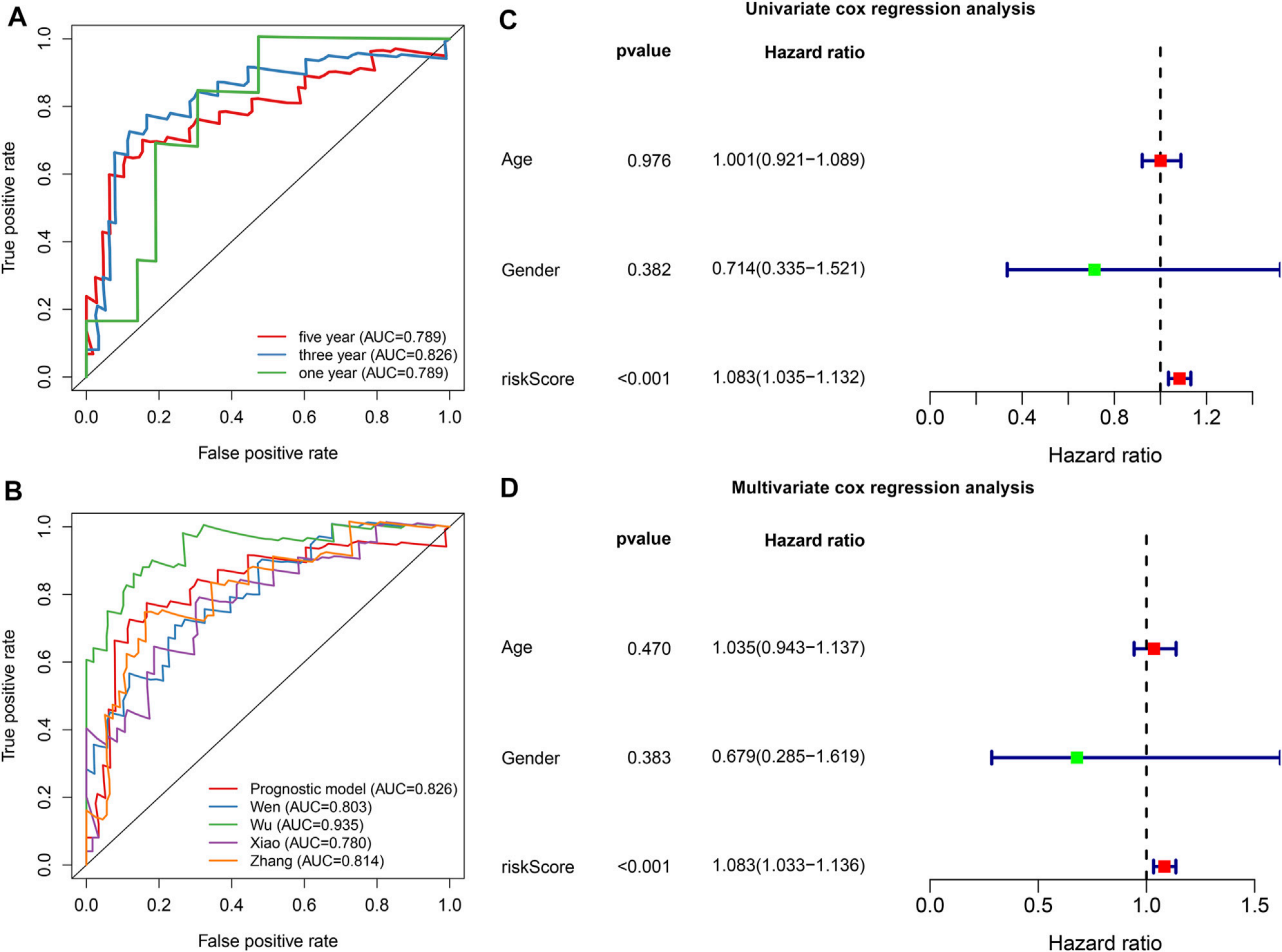
The hypoxic gene signature robustly and independently predicts osteosarcoma patients’ prognosis in the TARGET cohort. **(A)** Time-independent ROC curves of 1-, 3-, and 5-years survival based on risk score. **(B)** Comparisons of AUCs among different gene signatures for osteosarcoma patients. **(C, D)** Univariate and multivariate cox regression analyses for the associations between prognosis and age, gender, and risk scores.

To evaluate the predictive efficacy of this hypoxic gene signature on survival outcomes, time-independent ROC curves were conducted and the AUCs of 1-, 3-, and 5-years OS were 0.789, 0.826, and 0.789, respectively (Figure 3A). In comparison to other prognostic models, this signature had the higher accuracy and sensitivity for predicting prognosis of osteosarcoma patients (Figure 3B). Through univariate cox regression analyses, risk score displayed a significant association with osteosarcoma prognosis (*p* < 0.001, HR: 1.083, 95%CI: 1.035–1.132; Figure 3C). Multivariate cox regression analyses confirmed that risk score was independently predictive of clinical outcomes (*p* < 0.001, HR: 1.083, 95%CI: 1.033–1.136) with age, gender, and risk score as variables (Figure 3D).

### External Validation of the Predictive Performance of Hypoxic Gene Signature on Osteosarcoma Prognosis

**FIGURE 4:**
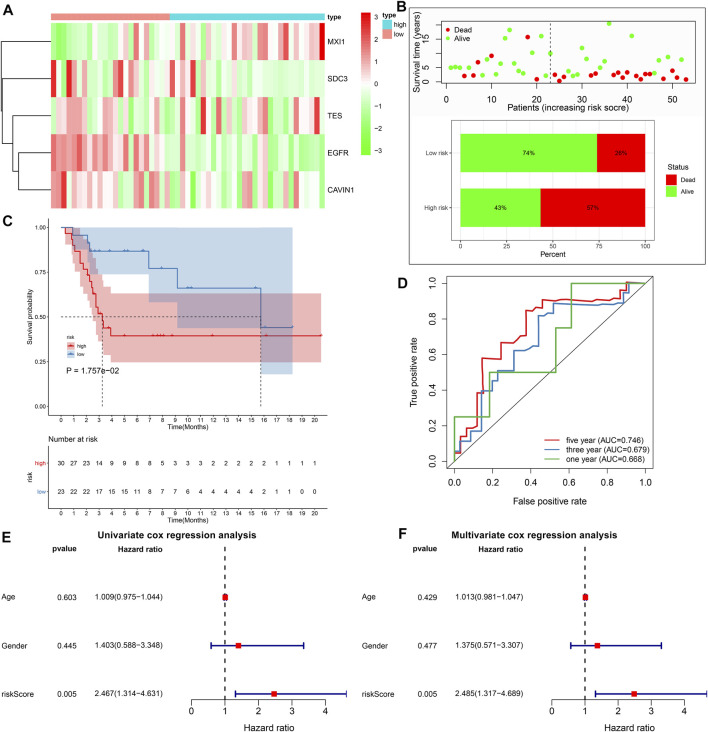
External validation of the predictive performance of hypoxic gene signature on osteosarcoma prognosis in the GSE21257 cohort. **(A)** Heat map visualizing the up- (red) and down-regulated (green) genes in high- and low-risk groups. **(B)** The distributions and proportions of survival status (alive: green; dead: red). **(C)** Kaplan-Meier curves of OS between high- and low-risk subjects. The differences were compared by log-rank tests. **(D)** Time-independent ROC curves of 1-, 3-, and 5-years survival based on risk score. **(E, F)** Univariate and multivariate cox regression analyses for the associations between prognosis and age, gender, and risk scores.

With the same formula, the risk score of each subject in the GSE21257 cohort was determined. Figure 4A depicted the expression of MXI1, SDC3, TES, EGFR, and CAVIN1 in high and low risk osteosarcoma specimens. The percentages of dead patients were 57 and 26% in the high- and low-risk groups, respectively (Figure 4B). Furthermore, unfavorable survival outcomes were found in high-risk subjects (*p* = 1.757e-02; Figure 4C), which exhibited the consistency with the results in the TARGET cohort. The AUCs of 1-, 3-, and 5-years survival were 0.668, 0.679, and 0.746, respectively (Figure 4D). Under univariate cox regression analyses, the risk score was in relation to desirable clinical outcomes of osteosarcoma (*p* = 0.005, HR = 2.467, and 95% CI: 1.314–4.631; Figure 4E). Nevertheless, age and gender were not significantly correlated to osteosarcoma prognosis. Multivariate cox regression analyses demonstrated that this signature was capable of independently predicting prognosis of osteosarcoma patients (*p* = 0.005, HR = 2.485, and 95% CI: 1.317–4.689; Figure 4F).

### Subgroup Analysis Identifies Prognostic Value of the Gene Signature in Osteosarcoma

**FIGURE 5:**
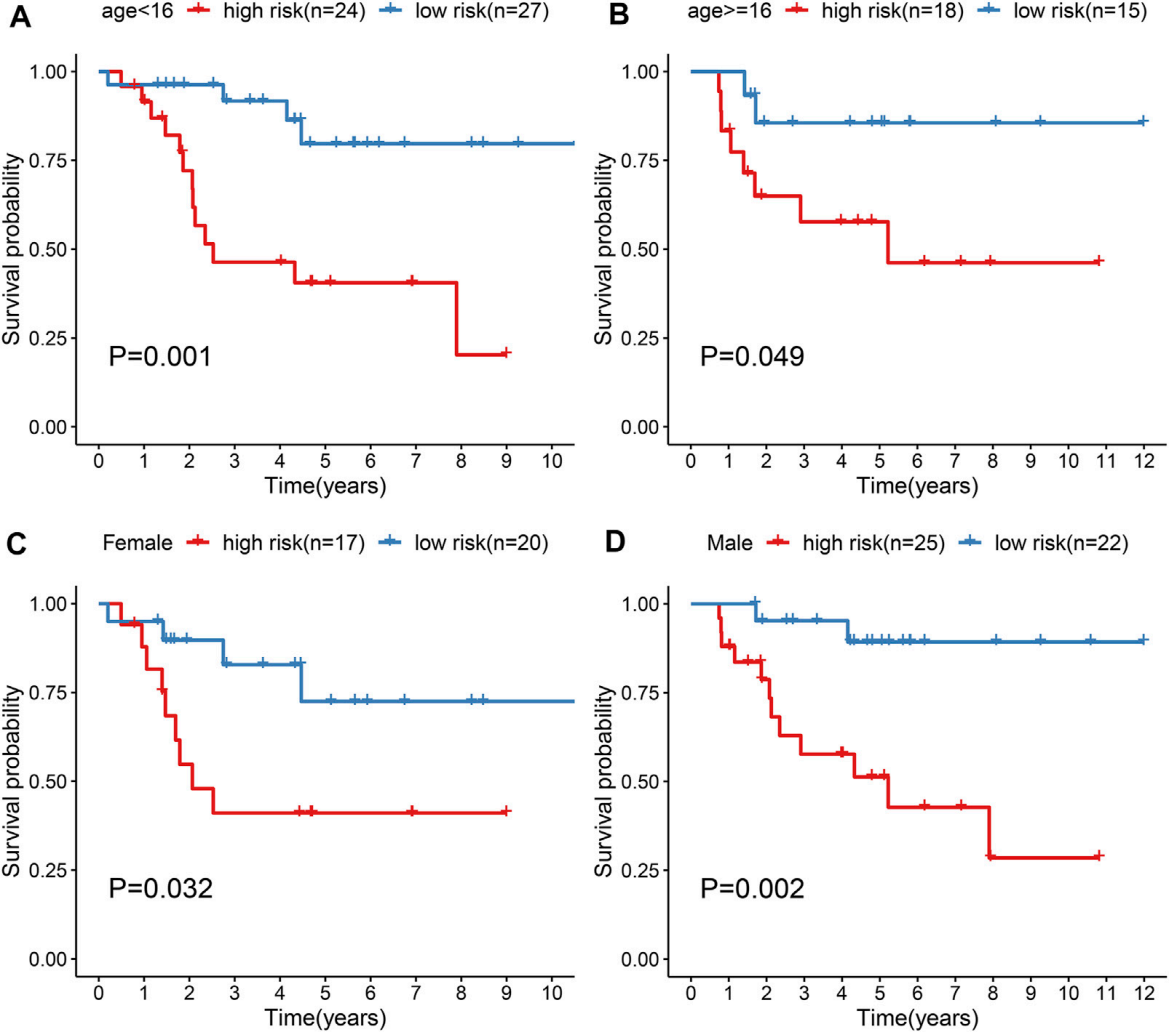
Evaluation of prognostic value of the gene signature in osteosarcoma by subgroup analysis. **(A, B**) Kapan-Meir curves of OS between high- and low-risk patients in **(A)** age <16 and **(B)** age ≥16 subgroups. **(C, D)** Kapan-Meir curves of OS between high- and low-risk patients in **(C)** female and **(D)** male subgroups. The survival differences were assessed by log-rank tests.

Subgroup analysis was applied to evaluate the predictive performance of the gene signature on osteosarcoma prognosis. First, patients from the TARGERT cohort were classified into age <16 and ≥16 subgroups. We found that high risk scores were in relation to worse survival duration both in two subgroups (*p* = 0.001 and 0.049; Figures 5A,B). Also, both in female (*p* = 0.032; Figure 5C) and male (*p* = 0.002; Figure 5D) subgroups, high-risk scores were indicative of shorter survival outcomes of osteosarcoma patients.

### GSEA Identifies Involved Signaling Pathways

**FIGURE 6:**
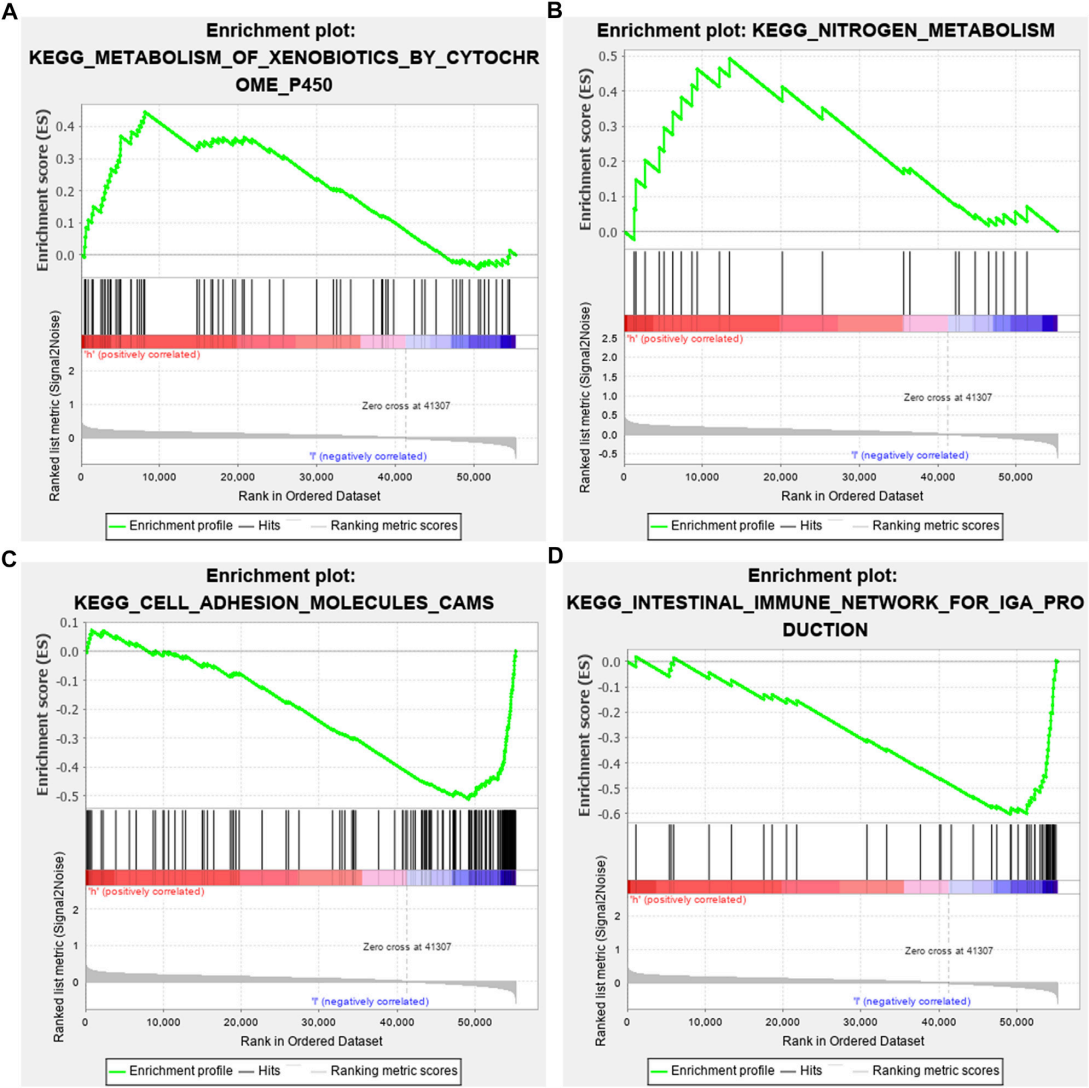
Identifying involved signaling pathways. **(A)** Metabolism of xenobiotics by cytochrome P450; **(B)** nitrogen metabolism; **(C)** cell adhesion molecules cams; and **(D)** intestinal immune network for IgA production.

GSEA results revealed that altered genes were distinctly enriched in several common pathways. Metabolism of xenobiotics by cytochrome P450 (NES = 1.56 and NOM *p*-value = 0.045) and nitrogen metabolism (NES = 1.52 and NOM *p*-value = 0.039) were mainly activated in the high-risk osteosarcoma samples (Figures 6A,B). In the meanwhile, low-risk samples were distinctly related to cell adhesion molecules cams (NES = -1.74 and NOM *p*-value = 0.035) and intestinal immune network for IgA production (NES = -1.78 and NOM *p*-value = 0.041; Figures 6C,D).

### Prognostic Values of Hypoxic Genes From the Gene Signature

**FIGURE 7:**
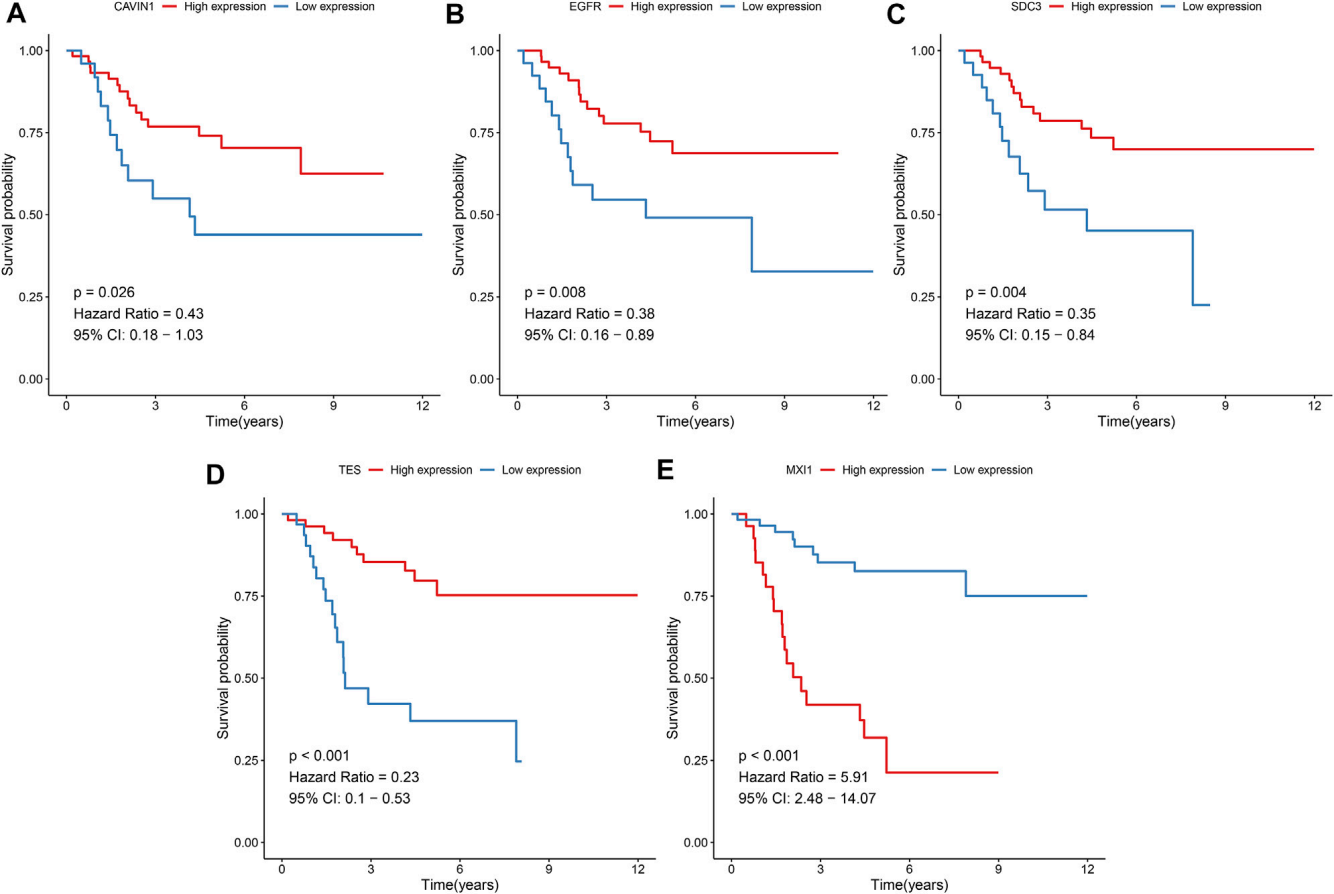
Prognostic values of hypoxic genes from the gene signature in osteosarcoma. Kaplan-Meier curves for overall survival of osteosarcoma patients in the high and low **(A)** CAVIN1, **(B)** EGFR, **(C)** SCD3, **(D)** TES, and **(E)** MXI1 expression groups from the TARGET cohort. The survival differences were assessed via log-rank tests.

The prognostic values of hypoxic genes from the gene signature were independently assessed in the TARGET cohort. As a result, high expression of CAVIN1 (*p* = 0.026, HR = 0.43, and 95% CI = 0.18–1.03), EGFR (*p* = 0.008, HR = 0.38, and 95% CI = 0.16–0.89), SCD3 (*p* = 0.004, HR = 0.35, and 95% CI = 0.15–0.84), and TES (*p* < 0.001, HR = 0.23, and 95% CI = 0.1–0.53) exhibited the prolonged survival duration of osteosarcoma patients (Figures 7A–D). Meanwhile, MXI1 up-regulation was in relation to unfavorable survival outcomes of osteosarcoma patients (*p* < 0.001, HR = 5.91, and 95% CI = 2.48–14.07; Figure 7E). Thus, each factor of the gene signature was associated to osteosarcoma prognosis.

### Differences in Immune Status Between High- and Low-Risk Osteosarcoma

**FIGURE 8:**
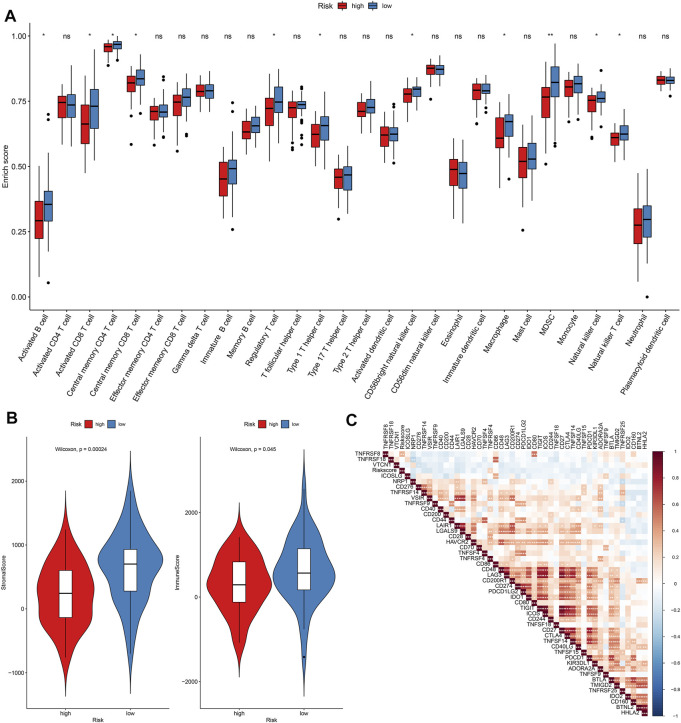
Assessment of differences in immune status between high- and low-risk osteosarcoma in the TARGET cohort. **(A)** Relative enrichment scores of immune cell subpopulations in high- and low-risk osteosarcoma samples. **(B)** Distributions of stromal and immune scores between high- and low-risk osteosarcoma samples. The differences were evaluated through Wilcoxon test. **(C)** Heatmap for the correlations between risk score and immune cell subpopulations in osteosarcoma. The darker the color, the stronger the correlation. Ns: not significant; *p < 005; **p < 0.01; ***p < 0.001.

The enrichment scores of immune cells were quantified in osteosarcoma specimens by applying the ssGSEA algorithm. Our data demonstrated that higher infiltrations of activated B cell s, activated CD8 T cells, central memory CD4 T cells, central memory CD8 T cells, regulatory T cells, type 1 T helper cells, CD56bright natural killer cells, macrophages, MDSC, natural killer cells, and natural killer T cells were found in the low-risk osteosarcoma in comparison to the high-risk osteosarcoma in the TARGET cohort (Figure 8A). Also, there were higher stromal scores (*p* = 0.00024) as well as immune scores (*p* = 0.045) in the low-risk specimens than the high-risk specimens (Figure 8B). The correlations between risk scores and immune checkpoints were assessed in osteosarcoma subjects. As a result, risk scores were positively correlated to TNFSF4 (Figure 8C).

### Correlations Between Risk Score and Sensitivity to Chemotherapy Drugs

**FIGURE 9:**
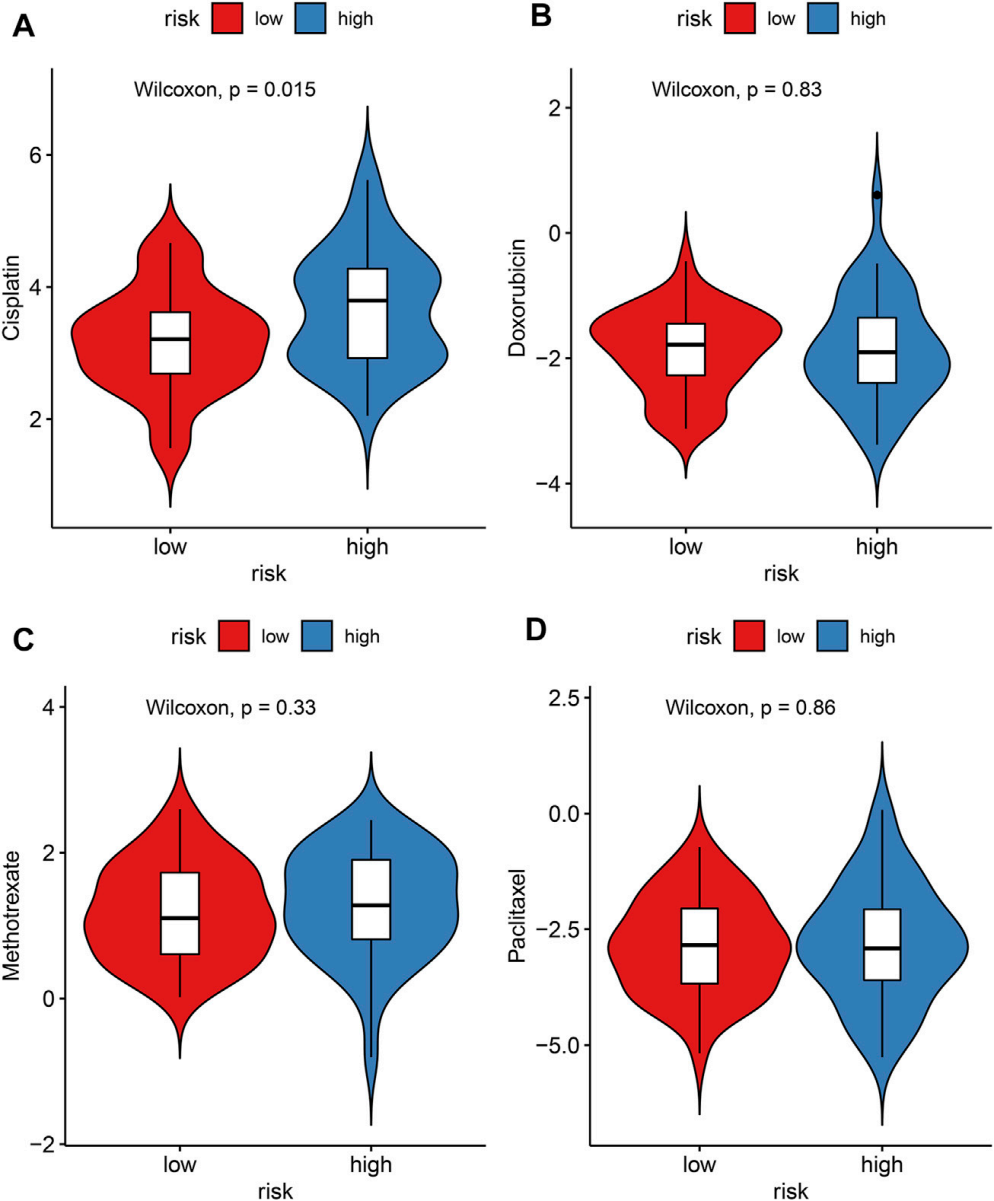
Evaluation of the correlations between risk score and sensitivity to chemotherapy drugs in the TARGET cohort. **(A)** Cisplatin; **(B)** doxorubicin; **(C)** methotrexate; **(D)** paclitaxel. The differences were assessed by Wilcoxon tests.

The IC50 values of chemotherapy drugs were determined in osteosarcoma patients from the TARGET cohort. Lowered IC50 values of cisplatin were detected in the high-risk compared to low-risk specimens (*p* = 0.015; Figure 9A), indicating that high-risk patients might be more sensitive to cisplatin. However, there were no significant differences in IC50 values of doxorubicin, methotrexate, and paclitaxel between groups (Figures 9B–D).

### Construction and Verification of a Personalized Prognostic Nomogram

**FIGURE 10:**
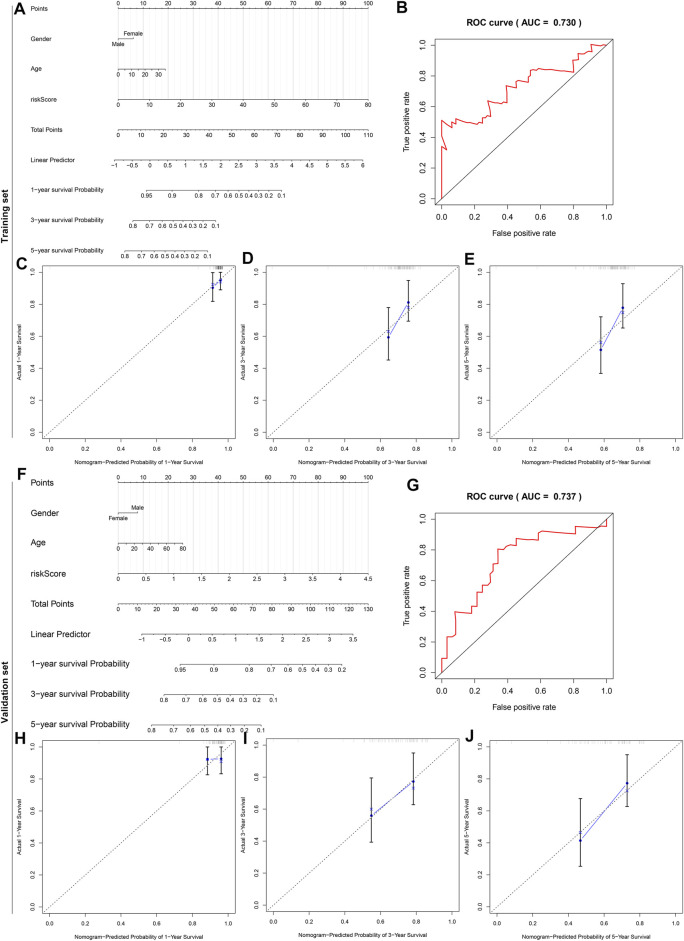
A nomogram for prediction of osteosarcoma patients’ prognosis. **(A)** A nomogram combining gender, age, and risk score for determining the 1-, 3-, and 5-years survival probability of osteosarcoma patients in the TARGET cohort. **(B)** The time-dependent ROC curves of this nomogram for overall survival of osteosarcoma patients. **(C–E)** The calibration plots of the nomogram for prediction of survival probability at 1, 3, and 5 years. The *x*-axis indicates the nomogram-predicted probability, and the *y*-axis indicates the actual survival. **(F)** External verification of the prognostic efficacy of this nomogram in predicting the 1-, 3-, and 5-years survival probability of osteosarcoma patients in the GSE21257 cohort. **(G)** The time-dependent ROC curves of the nomogram for 1-, 3-, and 5-years survival. **(H–J)** The calibration curves for the nomogram at 1, 3, and 5 years.

To offer an applicable quantitative tool for clinicians, a nomogram combining risk score, age, and gender was built for prediction of the probability of 1-, 3-, and 5-years survival of osteosarcoma patients in the TARGET cohort (Figure 10A). Each patient was determined based on each prognostic index. The higher number of total points was indicative of an undesirable prognosis for osteosarcoma patients. The AUC of OS time was 0.730, demonstrating that this nomogram exhibited high potential upon clinical utility (Figure 10B). Compared to the ideal model, the nomogram displayed a similar performance at 1-, 3-, and 5-years survival (Figures 10C–E). This prognostic nomogram model was externally verified in the GSE21257 osteosarcoma dataset (Figure 10F). The AUC value (0.737) showed that this nomogram possessed the favorable predictive capacity for the OS in osteosarcoma patients (Figure 10G). Our calibration curves showed excellent agreement between the nomogram prediction and actual observation at 1-, 3-, and 5-years survival in the GSE21257 dataset (Figures 10H–J). The above data suggested the appreciable reliability of this nomogram.

### Verification of the Expression of Hypoxic Genes From the Gene Signature

**FIGURE 11:**
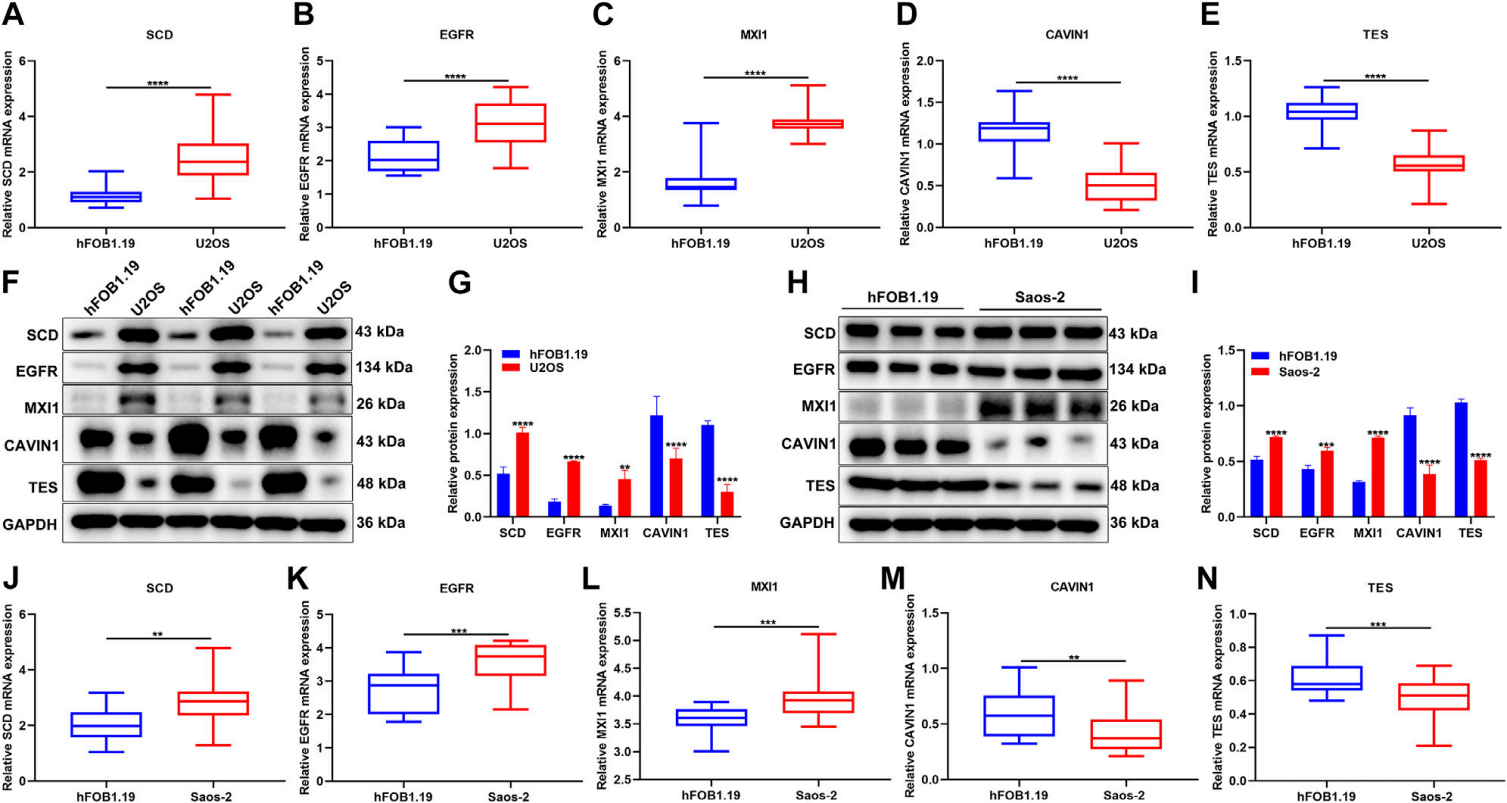
Verification of the expression of hypoxic genes from the gene signature. **(A–E)** RT-qPCR for detecting the expression of SCD3, EGFR, MXI1, CAVIN1, and TES in U2OS osteosarcoma cells and hFOB1.19 normal cells. **(F, G)** Western blotting for the expression of the above genes in U2OS osteosarcoma cells and hFOB1.19 normal cells. **(H, I)** Western blotting for the expression of the above genes in Saos-2 osteosarcoma cells and hFOB1.19 normal cells. **(J–N)** RT-qPCR for the expression of the above genes in tumors with Saos-2 osteosarcoma cells and hFOB1.19 normal cells. **p < 0.01; ***p < 0.001; ****p < 0.0001. U2OS osteosarcoma cells compared with hFOB1.19 normal cells.

The expression of hypoxic genes from the gene signature was verified in osteosarcoma and normal cell lines. Our results confirmed that SCD3, EGFR, and MXI1 were remarkably up-regulated while CAVIN1 and TES were prominently down-regulated in U2OS osteosarcoma cells compared with hFOB1.19 normal cells (Figures 11A–G). We also compared their expression in hFOB1.19 normal cells and Saos-2 osteosarcoma cells. Compared with hFOB1.19 cells, SCD3, EGFR, and MXI1 displayed higher expression while CAVIN1 and TES presented lower expression in Saos-2 cells, indicating that the above genes might participate in osteosarcoma progression (Figures 11H–N).

### Silencing MXI1 Attenuates Proliferation, Migration, and Invasion of Osteosarcoma Cells

**FIGURE 12:**
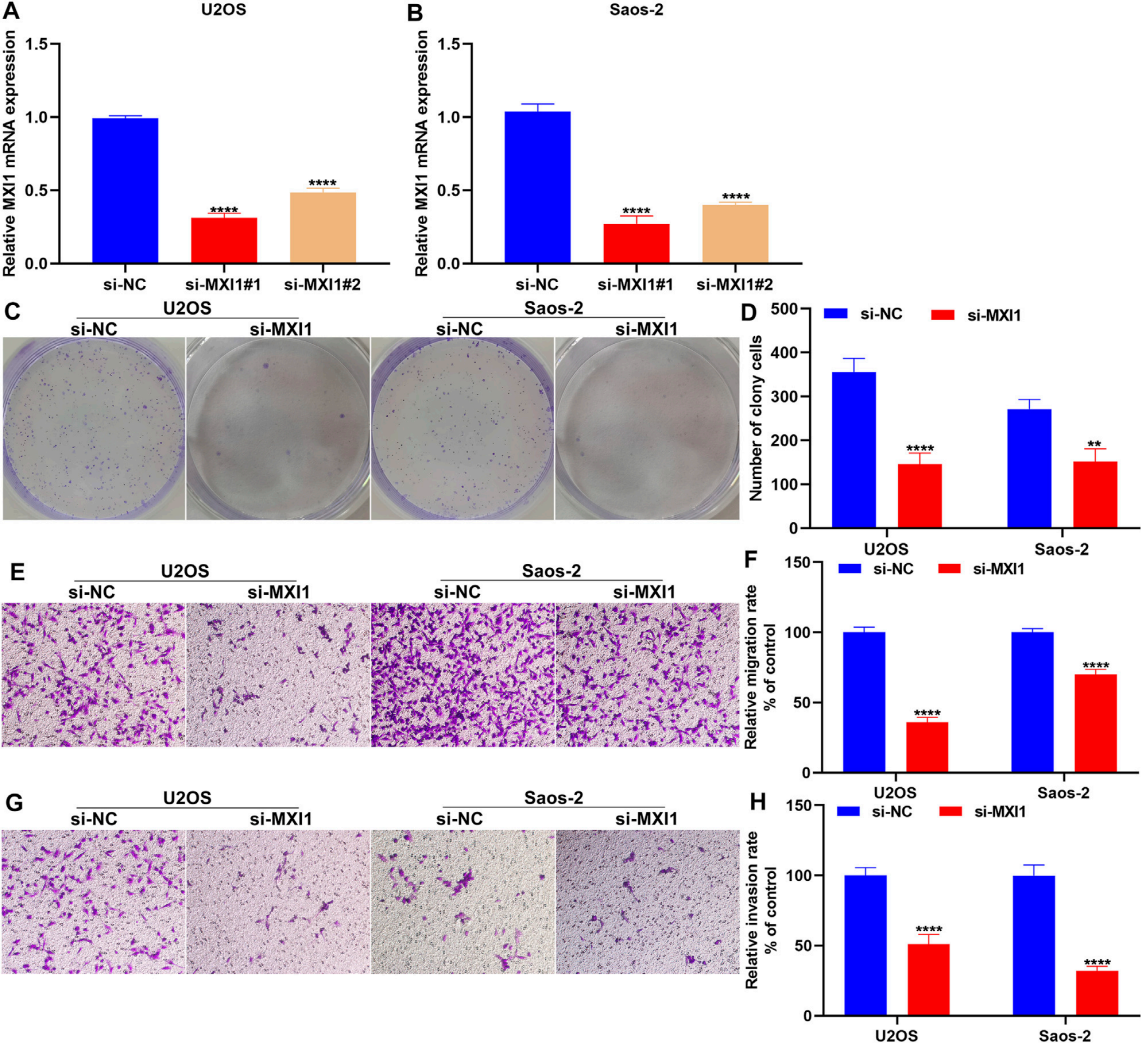
Silencing MXI1 attenuates proliferation, migration, and invasion of osteosarcoma cells. **(A, B)** RT-qPCR for verifying the expression of MXI1 in U2OS and Saos-2 cells following transfection with its specific siRNAs. **(C, D)** Colony formation for measuring the proliferative capacity of U2OS and Saos-2 cells after silencing MXI1 expression. **(E–H)** Transwell assays for detecting the **(E, F)** migration and **(G, H)** invasion of U2OS and Saos-2 cells with MXI1 knockdown. **p < 0.01; ****p < 0.0001.

The biological functions of MXI1 were further investigated through *in vitro* experiments. First, the expression of MXI1 was remarkably silenced by its specific siRNAs both in U2OS and Saos-2 cells (Figures 12A,B). Compared with the si-NC group, MXI1 knockdown prominently attenuated proliferation (Figures 12C,D), migration (Figures 12E,F), and invasion (Figures 12G,H) of U2OS and Saos-2 cells.

## Discussion

Hypoxia, a hallmark of cancer, may be induced by an insufficient oxygen supply, which is correlated to unfavorable survival outcomes, increased tumor heterogeneity, and drug resistance as well as immune escape (Vito et al., 2020). Here, we developed and verified a hypoxic gene signature for robustly predicting osteosarcoma prognoses, which could become a clinical stratified tool.

The hypoxic gene signature was built with the random survival forest algorithm, which consisted of CAVIN1, EGFR, SCD3, TES, and MXI1. High-risk scores were indicative of undesirable survival outcomes. Our ROCs confirmed the well predictive performance of this signature in osteosarcoma prognoses. The multivariate cox regression analyses were indicative of the predictive independency of the signature. Previously, a 28-gene hypoxic signature was proposed, which possessed the robust and independent prognostic efficacy in prostate cancer (Yang L. et al., 2018). Furthermore, a four-hypoxic gene model served as a prognostic factor for lung adenocarcinoma, which might predict the immunotherapeutic responses (Mo et al., 2020). The signaling pathways involved in risk scores were analyzed in osteosarcoma subjects. Metabolism of xenobiotics by cytochrome P450 and nitrogen metabolism were activated in the high-risk samples while cell adhesion molecules cams and intestinal immune network for IgA production were enriched in the low-risk samples. Evidence suggests that intestinal immune network for IgA production pathway is associated with cell proliferation and migration of hepatocellular carcinoma cells (Yang Z. et al., 2018). However, no study has reported the role of this pathway in osteosarcoma progression. Our study indicated that low-risk samples might have enhanced immune activation compared with high-risk samples. In the five-gene signature, we separately evaluated the prognostic potential of CAVIN1, EGFR, SCD3, TES, and MXI1 in osteosarcoma. Among them, up-regulation of CAVIN1, EGFR, SCD3, and TES was related to favorable survival outcomes while MXI1 up-regulation was associated with undesirable prognoses. Previously, CAVIN1 emerges as a novel tumor suppressor in Ewing sarcoma (Huertas-Martínez et al., 2017). Overactivity of EGFR is frequently observed in osteosarcoma and becomes an underlying therapeutic target (Ji and He, 2019). MXI1 is a key transcription factor involved in osteosarcoma progression (Xu et al., 2017). Additionally, our experimental results confirmed that SCD3, EGFR, and MXI1 were remarkably up-regulated while CAVIN1 and TES were prominently down-regulated in osteosarcoma cells compared with normal cells. The above findings highlighted the implications of these genes in osteosarcoma prognoses and progression. Our *in vitro* experiments demonstrated that silencing MXI1 attenuated proliferation, migration, and invasion of osteosarcoma cells, confirming the important role of MXI1 in osteosarcoma progression. Thus, our study is novel and better suited than previously published prognostic protocols for osteosarcoma (Wu Z.-l. et al., 2020; Qiu et al., 2021).

Cancer progression is highly correlated with the physiological state of osteosarcoma immune microenvironment that consists of innate as well as adaptive immune cells (Roma-Rodrigues et al., 2019). Osteosarcoma possesses a unique tumor microenvironment triggered by distinct molecular characteristics (Dyson et al., 2019). The immune microenvironment profiles are preponderant on prognoses and anti-tumor therapeutic efficacy. Immunotherapy emerges as a promising therapeutic strategy against osteosarcoma (Chen et al., 2021). Nevertheless, only minor subjects experience the desired anti-osteosarcoma immunity as well as impressive clinical response. For maximizing treatment effects of osteosarcoma subjects, it is a priority to alter the osteosarcoma immune microenvironment (Riera-Domingo et al., 2020). Hypoxia participates in anti-tumor immune response through lowering cytolytic and migratory activation of effector cells and the production and release of effector cytokines as well as triggering immunosuppressive cells, production and release of immunosuppressive cytokines, and expressions of immune checkpoint inhibitors (Multhoff and Vaupel, 2020). Herein, we found that there were higher infiltrations of activated B cells, activated CD8 T cells, central memory CD4 T cells, central memory CD8 T cells, regulatory T cells, type 1 T helper cells, CD56bright natural killer cells, macrophages, MDSC, natural killer cells, and natural killer T cells in the low-risk osteosarcoma than the high-risk osteosarcoma, indicating that this signature might reflect the immune microenvironment of osteosarcoma for bench observations. We noted that immunosuppressive cells and immuno-promoting cells were all significantly activated in low-risk groups, indicating the complex interactions between immunosuppressive cells and immuno-promoting cells during osteosarcoma progression. Infiltrating stromal and immune cells constitute the main fractions of the tumor microenvironment. Previous research has demonstrated that high stromal or immune scores indicated favorable survival outcomes of osteosarcoma (Hong et al., 2020). Their scores were determined in osteosarcoma tissues. The increase in stromal and immune scores was detected in the low-risk osteosarcoma specimens. Limited clinical activity of immune checkpoint inhibitors is investigated in osteosarcoma subjects (Wu C.-C. et al., 2020). Hence, it is of significance to gain the immunogenic potential of osteosarcoma. TNFRSF4 possesses potential as a target upon cancer immunotherapy (Buchan et al., 2018). Our data showed that risk score exhibited a positive correlation to immune checkpoint TNFRSF4 in osteosarcoma.

Chemotherapy resistance is the main issue in osteosarcoma therapy, which leads to undesirable prognoses (Prudowsky and Yustein, 2020). Alleviating hypoxia through tumor reoxygenation sensitizes the chemotherapy in osteosarcoma (Fu et al., 2021). Currently, there is a lack of clinically relevant molecular biomarkers that are predictive of the responses to chemotherapies. Cisplatin represents the first-line chemotherapy drug regarding osteosarcoma treatment, but chemoresistance limits the effectiveness of cisplatin (Wen J.-F. et al., 2020). Our data demonstrated that low-risk patients were more sensitive to cisplatin, indicating that this risk score might aid in overcoming cisplatin chemoresistance. The nomogram, as an appropriate clinical tool, has been widely utilized for quantitatively determining a person’s prognosis in the clinical setting through integration of several prognostic factors (Wu and Zhang, 2020). Here, the nomogram model combined risk score, age, and gender was established for predicting the probability of 1-, 3-, and 5-years survival time in osteosarcoma subjects. By calculating all the points, the nomogram yielded the numerical possibility of osteosarcoma patients concerning clinical outcomes. Following verification by ROCs and calibration plots, the nomogram model could be well predictive of 1-, 3-, and 5-years survival possibilities of osteosarcoma subjects. Nevertheless, the predictive efficacy of this nomogram requires to be verified in a larger osteosarcoma cohort.

## Conclusion

Collectively, we developed a hypoxic gene signature for risk stratification in osteosarcoma. After external verification, this risk score may be independently predictive of patients’ survival outcomes. Also, the risk score was in relation to osteosarcoma immune microenvironment and sensitivity to the chemotherapy drug cisplatin. A nomogram that combined the risk score, age, and gender became a promising prognostic classifier for osteosarcoma. Thus, our findings offered insights into personalized prognostication and therapy as well as follow-up scheduling.

## Data Availability

The original contributions presented in the study are included in the article/Supplementary Material, further inquiries can be directed to the corresponding authors.

## Abbreviations

AUC, area under the curve; CI, confidence interval; ESTIMATE, Estimation of STromal and Immune cells in MAlignant Tumours using Expression data; FDR, false discovery rate; GDSC, Genomics of Drug Sensitivity in Cancer; GSEA, gene set enrichment analysis; GEO, Gene Expression Omnibus; HR, hazard ratio; NES, normalized enrichment score; OS, overall survival; ROC, receiver operating characteristic; TARGET, Therapeutically Applicable Research to Generate Effective Treatments; ssGSEA, Single-Sample Gene Set Enrichment Analysis.
